# Structural Properties and Energy Band Alignment of Crystalline AlN Grown by Atomic Layer Deposition on Epitaxial Graphene

**DOI:** 10.3390/nano16110659

**Published:** 2026-05-24

**Authors:** Emanuela Schilirò, Salvatore Ethan Panasci, Raffaella Lo Nigro, Fabrizio Roccaforte, Blagoy Blagoev, Vladimir Mehandzhiev, Borislava Georgieva, Ivalina Avramova, Rositsa Yakimova, Milena Beshkova, Filippo Giannazzo

**Affiliations:** 1 Consiglio Nazionale delle Ricerche—Istituto per la Microelettronica e Microsistemi (CNR-IMM), Strada VIII 5, Zona Industriale, 95121 Catania, Italy; salvatoreethan.panasci@imm.cnr.it (S.E.P.); raffaella.lonigro@imm.cnr.it (R.L.N.); fabrizio.roccaforte@imm.cnr.it (F.R.); filippo.giannazzo@imm.cnr.it (F.G.); 2 Institute of Solid State Physics, Bulgarian Academy of Sciences, 72 Tzarigradsko Chaussee, 1784 Sofia, Bulgaria; blago@issp.bas.bg (B.B.); vmehandzhiev@issp.bas.bg (V.M.); 3 Institute of Electronics, Bulgarian Academy of Sciences, 72 Tzarigradsko Chaussee Blvd, 1784 Sofia, Bulgaria; b.georgiewa@abv.bg (B.G.); mbeshkova@yahoo.com (M.B.); 4 Institute of General and Inorganic Chemistry, Bulgarian Academy of Sciences, Acad. G. Bonchev Str. bl. 11, 1113 Sofia, Bulgaria; ivalina.avramova@gmail.com; 5 Department of Physics, Chemistry and Biology (IFM), Linköping University, SE-58183 Linköping, Sweden; rositsa.yakimova@liu.se

**Keywords:** atomic layer deposition, aluminum nitride, epitaxial graphene

## Abstract

In this work, the atomic layer deposition (ALD) of an ultra-thin AlN film on the surface of monolayer EG grown on-axis 4H-SiC(0001) substrates has been investigated as a function of the number of ALD cycles. The formation of a homogeneous film with a 10 nm thickness and crystalline wurtzite structure was obtained after 320 cycles, as demonstrated by atomic force microscopy (AFM) mapping, X-ray photoelectron spectroscopy (XPS) and X-ray diffraction. Raman mapping revealed a significant reduction in the native compressive strain of as-grown EG (ε ≈ −0.36%) with increasing ALD cycles, down to a value of −0.16% after full coverage. Finally, Kelvin Probe Force Microscopy (KPFM) surface potential mapping allowed the evaluation of energy band alignment of the AlN/EG heterojunction, with a conduction band offset of ~2.6 eV between the crystalline AlN film and the underlying EG. Such a large offset confirms AlN as a promising gate dielectric for EG-based devices.

## 1. Introduction

Epitaxial graphene (EG) grown via thermal decomposition of SiC (0001) is highly compelling because, unlike the CVD-grown graphene on metals, it can be directly used as-grown, without a transfer process, thereby avoiding risks of contamination and structural damage. The main challenge in EG growth is achieving uniform monolayer (1L) coverage across the entire SiC surface. However, under specific conditions of temperature and pressure, a highly uniform monolayer of EG (>98%) has been obtained on on-axis SiC (0001) [1]. Owing to its specific growth mechanism, mediated by the formation of an interfacial carbon buffer-layer partially sp^3^ hybridized with the Si-terminated face, EG exhibits a precise rotational alignment with the SiC substrate, compressive strain, and high n-type doping (10^13^ cm^−3^) [2]. Thanks to its excellent electronic transport properties, EG is highly promising for a variety of applications [3], including metrology [4], high-frequency electronics [5] and sensing [6].

Integrating thin insulating layers onto the graphene surface is essential for the fabrication of most graphene-based devices. Owing to its layer-by-layer growth mechanism, atomic layer deposition (ALD) represents the method of choice in microelectronics to deposit ultra-thin dielectric films with uniform and conformal coverage and sub-nanometer control of thickness [7,8]. Nevertheless, the chemically inert nature of the graphene sp^2^ lattice, characterized by a lack of dangling bonds, typically hinders the initiation of ALD growth [9,10]. Current strategies to achieve uniform deposition involve either functionalizing the graphene lattice or pre-depositing seed layers [11,12,13,14,15]. The functionalization process converts part of the sp^2^ bonds into out-of-plane sp^3^ bonds, providing the active sites needed for ALD nucleation, but it causes a partial degradation of graphene’s electrical properties, i.e., a reduction in the electron-mean-free path and carrier mobility. On the other hand, using seed layers (polymers, self-assembled monolayers, or metal/metal-oxide thin films) to promote the ALD process does not cause significant damage in the graphene lattice, but a poor interface quality of the insulating film with graphene is typically obtained, with the presence of defects responsible for charge trapping phenomena and hysteresis of the electrical characteristics. Furthermore, the interfacial seed layer leads to an increased equivalent oxide thickness (EOT), ultimately limiting gate insulator scalability for transistor applications. An alternative strategy to achieve nearly ideal insulator/graphene heterojunctions with very low interface state density is the adoption of van der Waals (vdW) bonded crystalline dielectrics, such as hexagonal boron nitride (h-BN), calcium fluoride (CaF_2_) or hexagonal aluminum nitride (h-AlN) [11,16,17]. In most cases, owing to their high deposition temperatures, these crystalline insulators are grown on foreign substrates and subsequently transferred onto 2D materials, resulting in significant damage and contamination related to the transfer process. However, recently, h-AlN has been grown on MoS_2_ via ALD at a temperature of 250 °C, and a vdW epitaxial interface between the two materials has been demonstrated [18,19]. AlN is particularly noteworthy among the crystalline dielectrics due to its wide bandgap (6.2 eV) and relatively high dielectric constant (κ ≈ 8), which make it an excellent candidate for gate dielectric applications. Additionally, being deposited via NH_3_ plasma, which does not damage the underlying 2D membrane, AlN could act as a protection layer prior to the plasma-enhanced ALD (PE-ALD) of oxides [20]. In this role, AlN also functions as an effective seed layer to improve the subsequent growth of high-k dielectrics.

In this work, we studied the direct ALD of AlN layers on EG on on-axis 4H-SiC. Specifically, we investigated the coverage degree, the quality of the deposited AlN film, and the resulting impact on the properties of graphene. For this purpose, the evolution of AlN coverage as a function of the number of ALD cycles (160 and 320) was analyzed via atomic force microscopy (AFM). Subsequently, the chemical properties and structural quality of the deposited AlN were evaluated through X-ray photoelectron spectroscopy (XPS) and X-ray diffraction (XRD), respectively. In addition, PeakForce Kelvin Probe Force Microscopy (PF-KPFM) was employed to characterize the surface potential of the AlN layer, and the conduction band offset between the AlN layer and the underlying EG was estimated. Finally, the effect of the AlN deposition on the structural properties of the EG was assessed by Raman spectroscopy, displaying a non-uniform compressively strained EG after 160 cycles and a decrease in the compressive strain on the sample with a complete AlN coverage (320 cycles). Such vibrational results are in perfect agreement with the previous KPFM analysis, confirming the evolution of AlN growth on EG as a function of the ALD cycles and demonstrating the beneficial impact of the integration of AlN on EG, from the electrical to the structural point of view.

## 2. Materials and Methods

The EG samples used for these experiments were obtained by thermal decomposition of nominally on-axis 4H-SiC (0001) at a temperature of 2000 °C in inert gas (Ar) at atmospheric pressure using an inductively heated sublimation reactor. By using specific well-controlled growth conditions (temperature distribution in the growth cell, temperature ramping up, and base pressure), a very uniform monolayer EG coverage on most of the SiC surface was obtained [21].

AlN depositions were carried out on the above-described EG using a Beneq TFS-200 reactor (Beneq Oy, Espoo, Finland) at 330 °C. Trimethylaluminium (TMA) and ammonia (NH_3_) were used as the aluminum precursor and nitrogen source, respectively. Each ALD cycle consisted of four stages: a 180 ms TMA dose, a 2 s N_2_ purge, a 90 ms NH_3_ dose, and a final 9 s N_2_ purge. Two samples were investigated, prepared with 160 and 320 ALD cycles.

Compositional analyses of the deposited AlN were carried out by X-ray photoelectron spectroscopy, in an Axis Supra electron spectrometer using achromatic AlKα radiation with an energy of 1486.6 eV. The analyzer pass energy used for survey spectra was 160 eV, while for the record of core level spectra, we used 20 eV. The recorded spectra were calibrated according to binding energies (BE) of the C 1 s line as a reference with an energy of 285.0 eV. The accuracy of the measured BE was 0.1 eV. The photoelectron lines of constituent elements on the surface were recorded and corrected by subtracting a Shirley-type background and quantified using the peak area and Scofield’s photoionization cross-sections. The deconvolution of spectra, where necessary, was performed with XPSPEAK41 software. A preliminary overview of XPS spectra of the samples after 160 and 320 ALD cycles allowed the identification of the surface chemical elements (Si, C, Al, N, O) and confirmed the absence of unwanted impurities, other than oxygen and adventitious carbon.

The AlN film crystallinity was evaluated by X-ray diffraction analysis using a Rigaku SmartLab XE 9 kW diffractometer (Rigaku Corporation, Akishima, Tokyo) by 2Theta/Omega scan measurements on the (0002) reflections.

AFM morphology and phase images of the EG samples before and after AlN deposition were carried out using a Bruker Icon Dimension system in PeakForce tapping mode with triangular tips (model PFQNE-AL by Bruker, MA, USA) featuring a nominal curvature radius of 5 nm and an oscillation frequency of 300 kHz. The same tips were employed in PeakForce KPFM mode for the evaluation of the contact potential difference between the AlN and EG. The work function of bare EG on 4H-SiC (WEG = 4.2 eV) was used for surface potential calibration. Furthermore, to extract statistically relevant information, surface potential values were evaluated by fitting the histograms extracted from KPFM maps (512 × 512 pixels) rather than from single scan lines. To ensure reproducibility of results, at least 5 KPFM maps were acquired on different regions for each sample.

Furthermore, nanoscale current-voltage characterization was performed by conductive atomic force microscopy (C-AFM) to evaluate the breakdown electric field, using conductive diamond-coated tips with a ≈5 nm curvature radius.

Micro-Raman measurements were carried out employing a Renishaw InVia spectrometer (Renishaw plc, Wotton-under-Edge, Regno Unito) equipped with a 100× objective and a 532 nm laser source, resulting in ~0.6 μm spot size focused on the sample surface. A ~5 mW incident power was used to avoid damaging the EG. A grating of 1800 L/mm was employed both for Raman single spectrum and mapping. Raman maps on bare EG and after 160 and 320 ALD cycles consisted of arrays of 10,201 spectra, collected on 50 μm × 50 μm areas with a grid spacing of 0.5 μm, and a total acquisition time of 20 min per acquisition map. For each spectrum in the map, the G and 2D peak frequencies were evaluated by fitting with a Gaussian function. The standard deviations of the peak maxima derived from the fits (±0.8 cm^−1^ for the G peak and ±1.8 cm^−1^ for the 2D peak) were taken as the uncertainty values. Statistical analyses of the 2D and G peak frequencies on these arrays of spectra allowed us to assess the impact of AlN deposition on the strain of monolayer EG.

## 3. Results and Discussion

**Figure 1:**
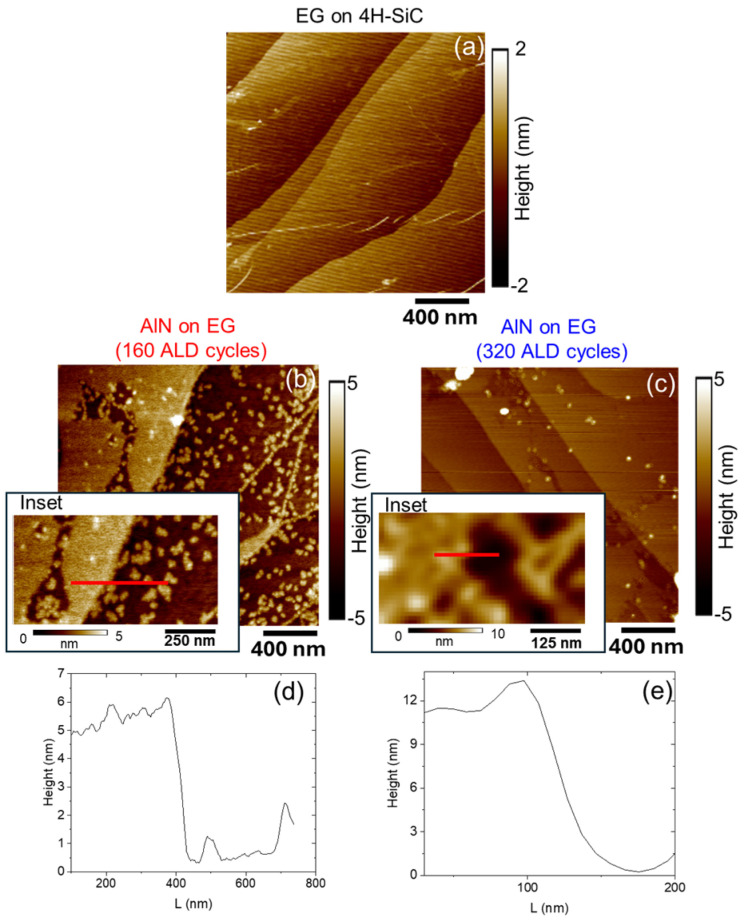
AFM morphology of as-grown EG on on-axis 4H-SiC (**a**) and after AlN deposition using 160 (**b**) and 320 (**c**) ALD cycles. Height linescan (**d**) from the red line of 160-cycle AlN inset in (**b**) and linescan (**e**) from the red line of 320-cycle AlN inset in (**c**).

Figure 1 shows the AFM surface morphologies acquired on as-grown EG on 4H-SiC (a) and after AlN deposition using 160 (b) and 320 ALD cycles (c), respectively. Representative height linescans extracted from the topography maps are also reported in Figure 1d,e. For as-grown EG samples (Figure 1a,d), graphene uniformly covers the micrometer-wide terraces and nanometer-high steps of the SiC substrate. Notably, while monolayer EG residing on (0001) terraces typically exhibits a dangling-bond-free sp^2^ lattice, a certain density of defects is concentrated at step edges. AFM analysis after 160 ALD cycles (Figure 1b) reveals an inhomogeneous AlN coverage of the EG surface, with the co-existence of sparse nanometric-sized islands nucleating on the sp^2^ EG terraces and continuous 2D-AlN regions formed by island coalescence starting from step edges. By measuring the height difference between the AlN-covered and uncovered regions (inset of Figure 1b), an AlN thickness of approximately 5 nm was evaluated (Figure 1d). On the other hand, after 320 ALD cycles (Figure 1c), a very uniform AlN surface coverage is achieved, with a deposited thickness of around 10 nm, evaluated by step height measurement (Figure 1e) on a scratch region of the AlN film reported as an inset of Figure 1c.

**Figure 2:**
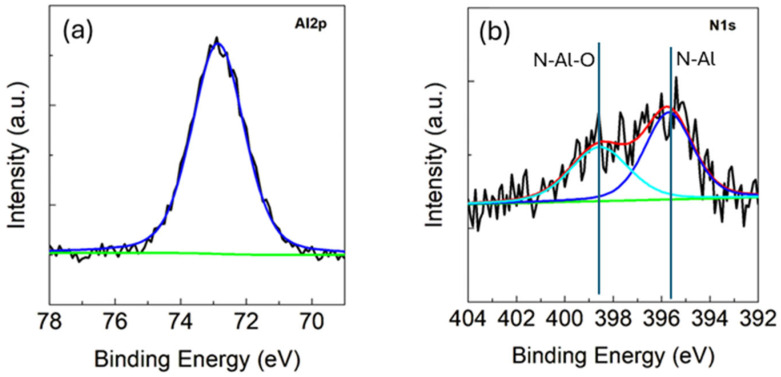
XPS spectra of AlN deposited on Epi-Gr/4H-SiC(0001). Al2p core-level line (**a**) and N1s core-level line (**b**). The blue is the peak refer to Al-N signal. Ciano is the peak refer to N-Al-O peak.

Given that the 320-ALD cycle process yields a homogeneous coverage, this sample was selected for further compositional and structural characterizations of the AlN layers. The deposition of AlN was first confirmed by X-ray photoelectron spectroscopy (XPS) analyses. Figure 2a displays the Al 2p core-level line at approximately 73.5 eV, which corresponds to the binding energy of AlN [22]. The N 1s core-level line, shown in Figure 2b, can be deconvoluted into two components at 396 and 398 eV, attributed to N-Al and N-Al-O bonds, respectively. The peak area ratio indicates that 55% is associated with Al-N and 45% with N-Al-O. AlN films deposited by ALD typically undergo surface oxidation upon air exposure. This is a well-known and documented phenomenon [23,24], which affects the first few atomic layers of the film down to a depth of approximately 5 nm [25]. Considering that we are investigating very thin AlN films of about 10 nm, this justifies the high intensity of the N-Al-O peak. This latter component indicates partial oxidation of the AlN film, consistent with previous works about ALD-grown AlN, typically reporting a significant oxygen incorporation, particularly in the surface region, because of ambient air exposure.

**Figure 3:**
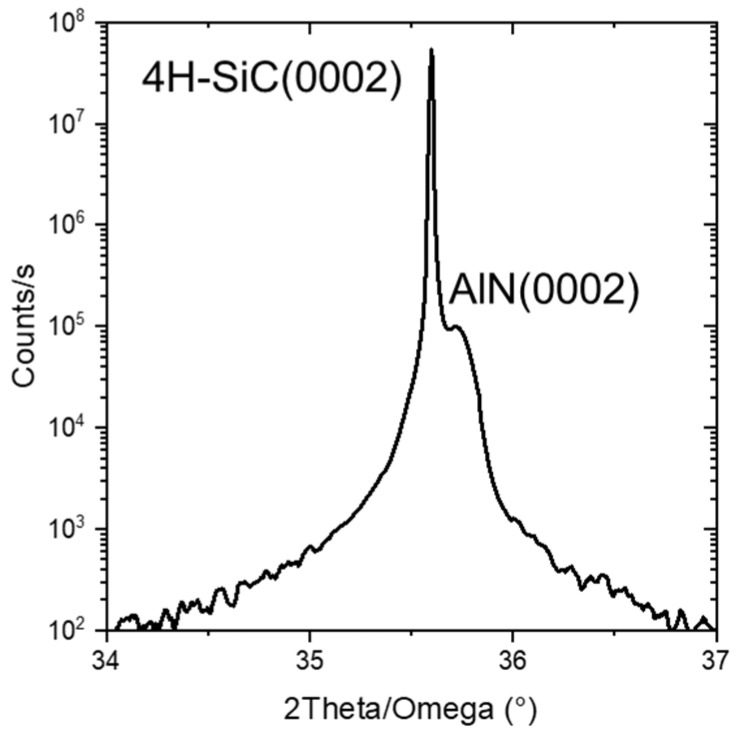
XRD 2Theta/Omega scan acquired on AlN films grown on EG/4H-SiC substrate by 320 ALD cycles.

The structural properties of the deposited AlN films were investigated by high-resolution XRD 2Theta/Omega scans, as reported in Figure 3. The very intense and narrow reflection at 35.5° corresponds to the (0002) planes of the 4H-SiC substrate, whereas the small peak at 35.7° can be attributed to the (0002) planes of AlN with wurtzite structure [26], indicating a crystalline structure of the 10 nm thick AlN film deposited by ALD onto EG.

**Figure 4:**
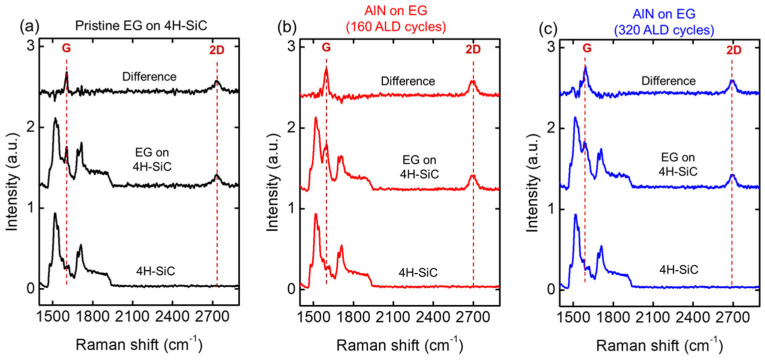
Raman spectra of pristine EG on 4H-SiC (**a**) and after AlN deposition with 160 (**b**) and 320 (**c**) ALD cycles. For each measured spectrum, the graphene signal has been extracted by subtraction of the 4H-SiC substrate Raman signal.

Raman spectroscopy was used to evaluate the impact of the AlN deposition on the structural properties (strain) of EG. Figure 4 shows three representative Raman spectra acquired on pristine EG on SiC (a), and after ALD growth of AlN using 160 (b) and 320 (c) ALD cycles. Since the characteristic vibrational features of graphene (i.e., the G peak and the 2D peak) are partially overlapped by the Raman signal of the SiC substrate, intensity normalization and subtraction of substrate components were applied in the three cases to retrieve the graphene signal. The results show that the 2D vs. G peaks intensity ratio is almost unaffected by the ALD.

**Figure 5:**
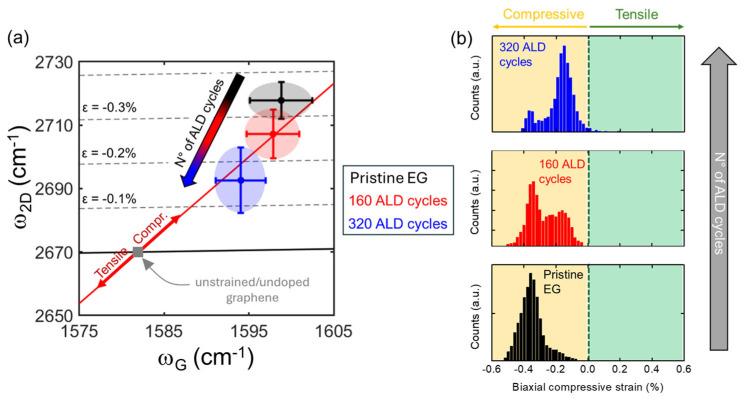
(**a**) Correlative ω_2D_ vs. ω_G_ plot showing the variation in the biaxial strain on the EG on 4H-SiC, before (black) and after AlN deposition using 160 (red) and 320 (blue) ALD cycles. (**b**) Distribution of the biaxial strain extracted from Raman maps collected on pristine EG (black) and after AlN growth with 160 (red) and 320 (blue) ALD cycles.

On the other hand, the 2D and G peak frequencies (ω_2D_ and ω_G_) exhibit significant shifts after the AlN deposition. This can be observed in the ω_2D_ vs. ω_G_ correlative plot reported in Figure 5a, where the black, red and blue ellipses represent the clouds of data-points extracted from arrays of 10,201 Raman spectra collected on the as-grown EG (reference) and after the 160 and 320 ALD cycles. This kind of plot can be used to quantitatively estimate the strain induced on the EG by the AlN deposition [27]. The red line is the ideal strain line, indicating the theoretical dependence of ω_2D_ on ω_G_ according to the graphene Grüneisen parameter. The gray square represents the literature values of ω_2D_ and ω_G_ for monolayer graphene unaffected by strain and doping (ω_2D,0_ = 1582 cm^−1^, ω_G,0_ = 2670 cm^−1^) [28]. Finally, the black line represents the semiempirical dependence of ω_2D_ on ω_G_ for n-type doped graphene. This analysis shows, for as-grown EG on 4H-SiC (black cloud), a significant biaxial compressive strain (>−0.3%), which is commonly ascribed to the thermal expansion coefficient mismatch between graphene and SiC during the cooling-down step after the high temperature growth. Interestingly, this compressive strain decreased to −0.27% (red cloud) after 160 AlN ALD cycles, and it reached a minimum of −0.16% (blue cloud) after the formation of a continuous AlN film using 320 ALD.

Notably, in a previous publication about thermal ALD of amorphous Al_2_O_3_ onto EG [27], an increase in compressive strain with deposited Al_2_O_3_ thickness was reported, i.e., an opposite trend with respect to the one observed in Figure 5a. Since thermal ALD processes for Al_2_O_3_ and AlN deposition involve similar thermal budgets, it can be excluded that heating effects or defect generation are responsible for the observed different trends in EG strain. On the contrary, this different behavior can be ascribed to the different nature of the EG interface with amorphous Al_2_O_3_ and crystalline AlN. In particular, it can be assumed that crystalline AlN grows epitaxially onto EG and introduces a tensile strain owing to its larger in-plane lattice constant (0.311 nm) with respect to graphene (0.246 nm). Hence, this tensile strain finally results in a compensation of the initial compressive strain of as-grown EG on 4H-SiC(0001).

The plot in Figure 5a provides information on the evolution of the average strain value and its standard deviation with the ALD process conditions. By applying the same procedure to each individual spectrum of Raman maps collected on the three samples, the distributions of graphene strain values can be evaluated, as illustrated in the histograms of Figure 5b. Notably, the strain distribution of pristine EG can be fitted by a main Gaussian peak centered at −0.36% and a very small contribution at lower compressive strain. After the 160 ALD cycles, the graphene strain distribution significantly changed, showing a bimodal shape, with a contribution aligned with the main peak of the bare EG and the second at lower compressive strain (−0.2%). Conversely, after 320 ALD cycles (resulting in a complete AlN coverage), the graphene strain distribution showed a main peak at −0.2%, with a residual contribution at higher compressive strain (−0.36%).

**Figure 6:**
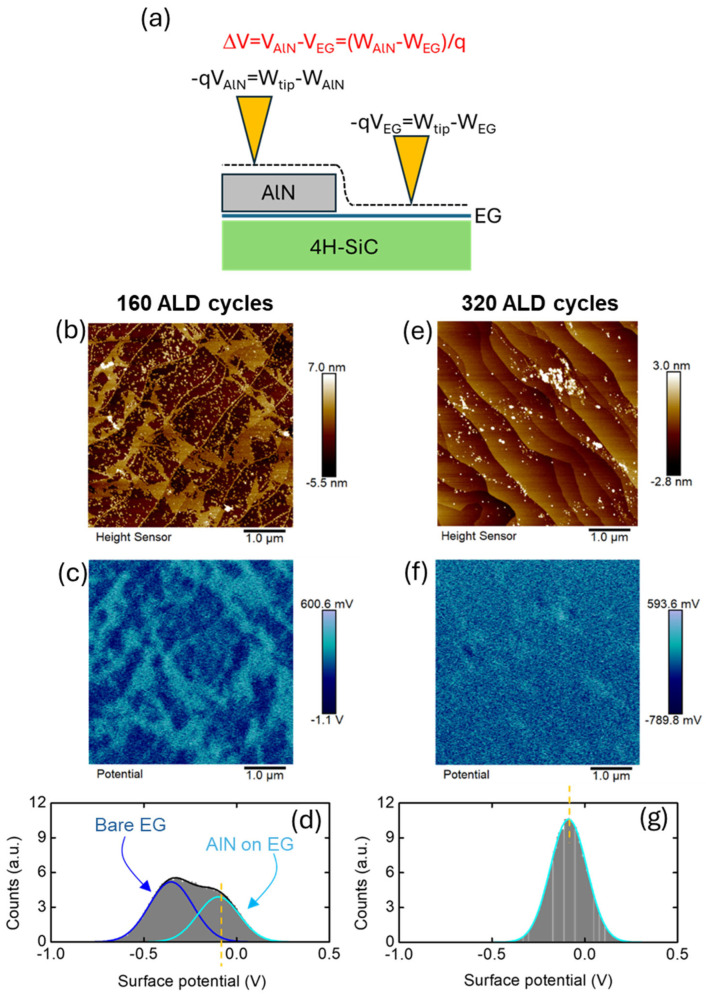
(**a**) Schematic illustration of the KPFM setup for the measurement of the surface potential on AlN-covered and bare EG regions. (**b**) Morphology, (**c**) surface potential map and (**d**) histogram of the surface potential distribution after 160 ALD cycles. (**e**) Morphology, (**f**) surface potential map and (**g**) histogram of the surface potential distribution after 320 ALD cycles.

Finally, the electronic properties of the ALD-grown crystalline AlN on EG were evaluated using Kelvin Probe Force Microscopy (KPFM). Figure 6a schematically illustrates a conductive tip with work function W_tip_, scanned in lift-mode on an EG region partially covered by AlN. During the scan, the local surface potential

V_AlN_ = −(W_tip_ − W_AlN_)/q (1)

and

V_EG_ = −(W_tip_ − W_EG_)/q (2)

are measured in the two regions, where W_tip_, W_AlN_ and W_EG_ are the tip, AlN and EG work functions, respectively. Hence, the surface potential difference between AlN and bare EG is related to the work function difference between the two materials:

ΔV = (W_AlN_ − W_EG_)/q. (3)

Figure 6b,c show the morphology and the corresponding KPFM surface potential map measured on the sample with AlN deposited by 160 ALD cycles. Notably, the surface potential map in Figure 6c exhibits clear differences between the potential signal associated with the AlN-covered (cyan regions) and uncovered (blue regions) EG/SiC. In contrast, after 320 ALD cycles, the surface potential map shown in Figure 6f exhibits a nearly uniform contrast, consistent with the uniform AlN coverage deduced by the corresponding morphological image in Figure 6e. The histograms extracted from the two surface potential maps are reported in Figure 6d and Figure 6g, respectively. For the 160-cycle sample, the surface potential distribution was fitted by two distinct Gaussian components at −0.34 V and −0.10 V, corresponding to the uncovered and AlN-covered EG areas, respectively. In contrast, the surface potential histogram for the 320 ALD-cycles sample (Figure 6g) was fitted by a single Gaussian component centered at −0.10 V, associated with the continuous AlN-covered surface.

**Figure 7:**
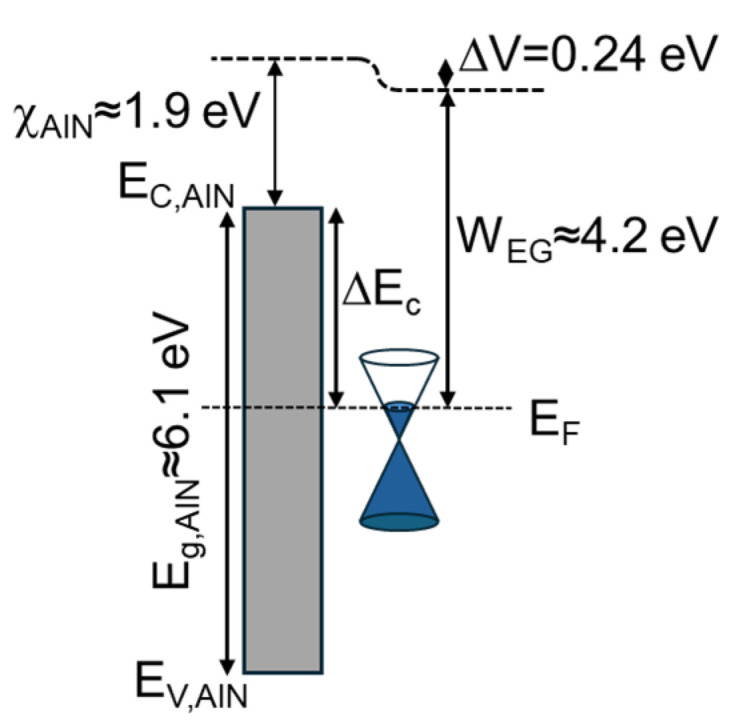
Energy band diagram deduced from the measured surface potential difference ΔV between AlN and bare EG.

According to Equation (3), the surface potential difference ΔV = 0.24 V between the two components in Figure 6d corresponds to the work function difference between deposited AlN and EG. From this experimental result, the energy band diagram of the AlN/EG heterojunction can be evaluated, as illustrated in Figure 7. Here, W_EG_ ≈ 4.2 eV is the typical work function for the highly n-type doped (10^13^ cm^−2^) EG on 4H-SiC(0001) [29], which is lower than the work function value of 4.5–4.6 eV for pristine monolayer graphene (with the Fermi level located at the Dirac point). Assuming the most common literature value for AlN electron affinity χ_AlN_ ≈ 1.9 eV [30], a conduction band offset of ΔE_C_ ≈ 2.5–2.6 eV can be deduced at the AlN/EG interface. As a matter of fact, oxygen incorporation in the ALD-grown AlN film, revealed by XPS analyses, is expected to affect both its energy bandgap and electron affinity. According to literature reports, AlON thin films typically exhibit a bandgap of ~6 eV, comparable to that of AlN. Furthermore, depending on the oxygen content and its distribution within the thickness of our 10 nm thick films, we can assume an intermediate electron affinity value between pure AlN (~1.9 eV) and Al_2_O_3_ (~2.6 eV). Properly modifying the energy band diagram of Figure 7 to account for the larger electron affinity of AlON would result in a reduced ΔE_C_ with respect to the case of pure AlN. In any case, the estimated band offsets based on these band alignment considerations are sufficiently large to consider AlN as a promising gate dielectric for EG-based transistors.

**Figure 8:**
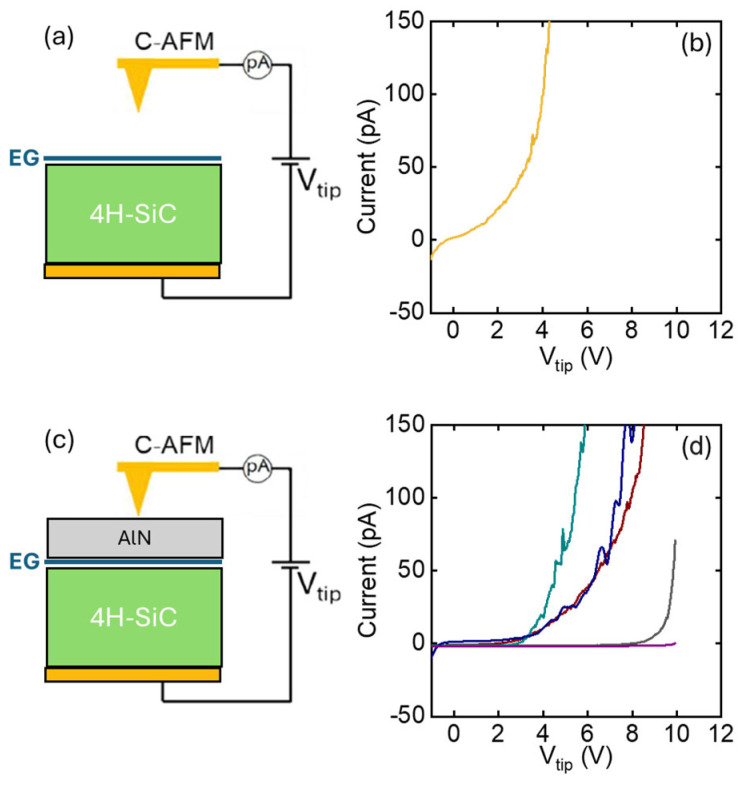
Schematic of the experimental setup C-AFM for local I-V measurements on bare EG/SiC (**a**) and on AlN/EG/SiC (**c**). I-V_tip_ characteristics for bare EG/SiC (**b**) and 320 ALD cycles of AlN on EG/4H-SiC (**d**).

However, current-voltage characterization is required to evaluate the insulation properties of AlN on EG. In this respect, the CAFM technique enables nanoscale-resolution current-voltage (I-V_tip_) analyses directly on the AlN surface, without requiring device fabrication processing that could potentially affect the film’s quality. To this purpose, front-to-back I-V_tip_ curves were acquired on the bare EG on SiC, and after the growth of the 10 nm AlN film, according to the configurations schematically illustrated in Figure 8a and Figure 8c, respectively. As compared to the representative I-V_tip_ characteristic on the bare EG/SiC sample (Figure 8b), which shows a current flow starting from V_tip_ = 0, most of the representative curves acquired at different positions on the AlN/EG/SiC sample exhibit a negligible leakage current up to ~4 V, followed by a gradual rise at larger voltages. On some positions, the leakage current remains negligible up to 8–10 V, followed by an abrupt increase in the conduction, which can be ascribed to a dielectric breakdown event. This variability can be correlated with the polycrystalline nature of the AlN film, where randomly distributed grain boundaries act as conduction paths, probably responsible for the gradual leakage increase above 4 V. Breakdown phenomena are observed in the cases when the nanoscale tip contact is far enough from grain boundaries.

## 4. Conclusions

In conclusion, we investigated the ALD deposition of AlN thin films on the surface of monolayer EG grown on on-axis 4H-SiC(0001) substrates. The evolution of AlN coverage was evaluated as a function of the number of ALD cycles, showing the formation of a homogeneous film with 10 nm thickness after 320 cycles. XPS confirmed the formation of AlN, with a partial oxidation of the surface due to ambient air exposure, as typically reported for ALD-grown films. Structural characterization via HR-XRD demonstrated that the AlN film possessed a well-oriented (0001) crystalline wurtzite structure. Raman mapping revealed a significant reduction in the native compressive strain of as-grown EG (ε ≈ −0.36%) with an increasing number of ALD cycles, down to a value of −0.16% after full coverage. This strain reduction, attributed to the in-plane lattice mismatch between crystalline AlN and EG, is expected to be beneficial for the electron mobility of graphene field-effect transistors. KPFM surface potential mapping enabled the evaluation of the energy band alignment of the AlN/EG heterojunction, revealing a conduction band offset of ~2.6 eV between the crystalline AlN film and the underlying EG. Finally, CAFM-based nanoscale-resolution current-voltage analyses on the 10 nm AlN/EG/SiC sample indicated good insulating properties of the AlN film up to V_tip_ = 4 V, followed by a gradual increase in the leakage current at higher voltages, which was associated with the grain boundaries of the crystalline AlN. Therefore, despite the positive effect on EG strain reduction and the favorable conduction band offset, the crystalline nature of the deposited AlN adversely affects its insulating properties. A possible strategy to enhance the insulating quality of the films could be the overgrowth of an amorphous dielectric capping layer, such as Al_2_O_3_, on top via ALD.

## Data Availability

The original contributions presented in this study are included in the article. Further inquiries can be directed to the corresponding author.
